# Dynamic Supramolecular Polyurethane Elastomers Enabling Bioinspired Strain‐Adaptive Stiffening to Resolve the Softness–Strength Paradox

**DOI:** 10.1002/advs.74910

**Published:** 2026-03-20

**Authors:** Haoming Zou, Zhi‐Xiong Fei, Zhao Yang, Ke‐Ke Yang, Dong‐Xu Yang, Ling‐Ying Shi

**Affiliations:** ^1^ College of Polymer Science and Engineering State Key Laboratory of Polymer Materials Engineering Sichuan University Chengdu China; ^2^ The Collaborative Innovation Center For Eco‐Friendly and Fire‐Safety Polymeric Materials (MoE) College of Chemistry Sichuan University Chengdu China; ^3^ State Key Laboratory of Optical Field Manipulation Science and Technology Institute of Optics and Electronics Chinese Academy of Sciences Chengdu China

**Keywords:** artificial skin, quadruple hydrogen bonding, self‐healing, strain‐adaptive stiffening, supramolecular polyurethane

## Abstract

The rapid progress of soft robotics and flexible electronics has sparked tremendous demand for elastomers that unite high mechanical strength with intrinsic softness, thereby mimicking the mechanical performance of natural skin. Herein, we report a class of dynamically crosslinked supramolecular polyurethanes that combine skin‐comparable low modulus with high strength and room‐temperature self‐healing, achieved through a segmental molecular engineering strategy. The design leverages entropy‐driven soft elasticity together with enthalpy‐governed strain‐stiffening and energy dissipation, dictated by polymer segment topology. Using a prototypical supramolecular elastomer, we demonstrate that incorporating an isophorone diisocyanate (IPDI) chain extender, whose steric configuration matches that of ureidopyimidinone (UPy), reinforces hydrogen bonding interaction and suppresses premature crystallization. The optimized elastomer exhibits an ultralow initial modulus (<3 MPa) yet undergoes pronounced strain‐stiffening, yielding exceptional tensile strength (>50 MPa) and high fracture toughness (∼2×10^5^ J m^−2^), outperforming state‐of‐the‐art room‐temperature self‐healing elastomers. Moreover, the elastomer features hydrophobicity, ensuring stability in underwater applications. These results underscore the promise of segment‐level molecular engineering for constructing dynamic polyurethanes with thermoplastic processability, skin‐like softness, and unprecedented mechanical robustness.

## Graphical abstract

Segmental‐level molecular engineering of a supramolecular polyurethane elastomer integrates strain‐adaptive chains, soft elastic segments, and dynamic hydrogen bonding motifs. This design reconciles skin‐like softness with muscle‐level strength, offering a sustainable and high‐performance material platform for next‐generation artificial skin and soft robotics.

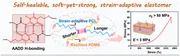

## 1 Introduction

Natural skins generally exhibit a strain‐adaptive stiffening phenomenon, where they are initially soft with low modulus to accommodate body motion, yet rapidly stiffen upon stretching to increase their strength by several orders of magnitude [1, 2]. This nonlinear mechanical performance imparts remarkable damage resistance and helps the skin to actively prevent injury under external stress [3]. The growing demand for artificial skin in emerging fields such as flexible electronics and soft robotics [4, 5, 6, 7] has sparked tremendous interest in the development of strain‐stiffening elastomeric materials that emulate the mechanical performance of natural skin.

To realize strain‐stiffening in soft elastomers, a variety of molecular‐ and macro‐scale design strategies have been developed, exploiting fundamental mechanisms such as polymer chains alignment, entropy restriction of network, crystallization of semicrystalline segments, and stress‐dependent dissociation‐reformation of dynamic bonds. Representative examples include liquid crystalline elastomers [8], chameleon‐like elastomers engineered from bottlebrush block copolymers [9], module‐assembled elastomer [10], mechanically trained hydrogels [11], and stepped‐modulus composites [12]. However, most reported elastomers either exhibit both low modulus and low strength (on the order of kPa [9]) or concurrently exhibit high modulus and high strength, even with the modulus value far exceeding the strength value [13, 14, 15]. Achieving softness (low Yang's modulus) yet high strength along with efficient room‐temperature self‐healing within an elastomer, therefore, remains a significant challenge.

Inspired by the reinforcing mechanisms of biological tissues that utilize dynamic sacrificial bonds [16], extensive efforts have been devoted to integrating coordination and hydrogen bonding into polyurethane elastomers to enhance the mechanical robustness [17, 18, 19, 20, 21, 22]. Among various dynamic interaction motifs [23, 24], ureidopyimidinone (UPy), a self‐complementary quadruple hydrogen‐bonding unit pioneered by Meijer and coworkers [25, 26], has emerged as a versatile building block for supramolecular network construction [27], owing to its high dimerization constant (*K*_dim_ > 10^7^ M ^−1^ ), strong binding affinity, inherent self‐complementarity, and synthetic accessibility. Incorporation of UPy units into main chain [19], side chain [28, 29], or chain termini [30, 31] of polymers has afforded materials with enhanced mechanical strength, reprocessability, and self‐healing capability. However, conventional design paradigms of elastomer with dynamic interaction design paradigms still face a trade‐off between softness and strength. For instance, Bao and co‐workers developed self‐healable, highly stretchable supramolecular poly(tetramethylene ether glycol) (PTMEG) and polydimethylsiloxane (PDMS) based supramolecular polyurethane elastomers (SPUE) by incorporating UPy motifs, though their tensile strength remained below 5 MPa [19, 21]. To meet the stringent demands of soft robotics and artificial skin, it is essential that elastomers simultaneously exhibit tactile softness and exceptional strength to ensure long‐term durability. The combination of multiple hydrogen bonding interactions within the strain‐stiffening supramolecular polyurethane systems may achieve the concurrent improvement of softness and strength, thereby fulfilling the demands of artificial skin.

**FIGURE 1:**
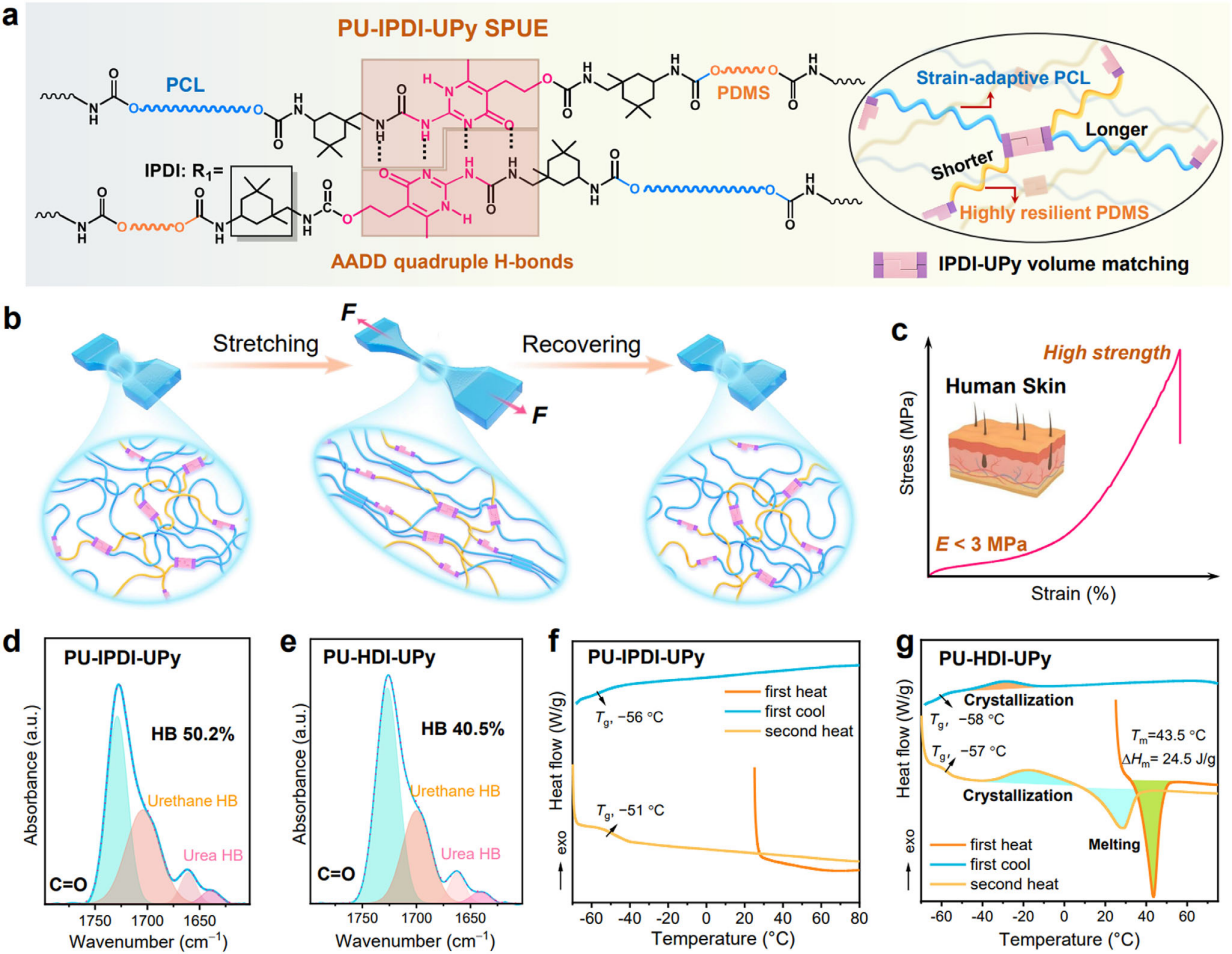
(a) Schematic diagram of the molecular design of multiblock PU‐IPDI‐UPy SPUE with the illustration of distinct structural roles of individual polymer segments. (b) illustration of the strain‐stiffening behavior of PU, attributed to reversible strain‐induced microcrystallization of the PCL segments. (c) The strain‐stiffening mechanical behavior of the artificial skin is analogous to that of natural skin. (d, e) FI‐IR spectra in the C═O absorption region (deconvoluted into peaks at 1730, 1700, 1660, and 1640 cm^−1^) and (f, g) DSC curves of PU‐IPDI‐UPy and PU‐HDI‐UPy, respectively.

Herein, we report a supramolecular polyurethane elastomer that emulates the strain‐adaptive stiffening of natural skin by integrating entropy‐governed elasticity with enthalpy‐driven sacrificial interactions (Figure 1a). Supersoft PDMS backbones endowed the network with low initial modulus, while polycaprolactone (PCL) segments underwent reversible strain‐induced crystallization to impart pronounced stiffening under tension. Concurrently, UPy nanodomains acted as dynamic crosslinkers, dissipating energy and enabling efficient self‐healing at room temperature. The mechanical behavior of the materials was systematically investigated and optimized by studying the effects of isocyanate topology and hydrogen‐bonding interactions through strategic substitution of isocyanates and chain extenders. As a result, the targeted elastomers combined skin‐like softness (low Young's modulus) with exceptional tensile strength, exhibiting a tensile strength‐to‐modulus ratio exceeding 17. In situ x‐ray diffraction analysis further unveiled the microphase evolution during deformation, elucidating the cooperative contributions of entropic and enthalpic mechanisms. This work establishes a segmental topology design strategy that reconciles softness and robustness in supramolecular elastomers and opens new opportunities for artificial skin, soft robotics, and other emerging flexible devices.

## 2 Results and Discussion

### 2.1 Molecular Design, Synthesis, and Characterization of SPUE

We designed a supramolecular polyurethane elastomer (SPUE) exhibiting strain‐adaptive stiffening behavior, which integrates low‐modulus, high‐strength, and self‐healing through segmental molecular engineering. As illustrated in Figure 1a, the SPUE design is guided by two key principles. (1) Volume‐matched chain extenders, IPDI and UPy, facilitate the formation of hydrogen bonding while suppressing PCL crystallization, and thus soft and amorphous polymer chains ensure low Yang's modulus and high‐level stretchability. (2) The incorporation of PCL segments endows the network with reversible strain‐adaptive stiffening and enhanced tensile strength, whereas the shorter PDMS segments promote elastomeric resilience by leveraging entropic elasticity arising from supramolecular cross‐linking and chain entanglement (Figure 1a,b). Such a molecular design gives rise to a soft and tough elastomer with low modulus and high strength (Figure 1c) that follows the non‐linear mechanical behavior of natural skin.

A two‐step chain‐extension process was employed to couple longer strain‐adaptive crystallizable PCL (*M*_n_, ∼2000 g mol^−1^) and shorter resilient PDMS block (*M*_n_, ∼500 g mol^−1^) via UPy and isocyanate chain extenders, yielding the PCL‐PDMS SPUE elastomer (Scheme S1). Briefly, isocyanate‐terminated PDMS and PCL prepolymers were first synthesized through the reaction of IPDI (alternative HDI for comparison) with a mixture of HO‐PCL‐OH and HO‐PDMS‐OH [32]. Here, IPDI features asymmetric substitution and a cyclic structure, whereas HDI is linear; these structural differences will affect the hydrogen bonding interactions and crystallization behavior of PCL segments. Subsequently, UPy chain extenders were sequentially introduced into the reaction system to synthesize PU. The molar ratio of PDMS to PCL was fixed at 1:4, and the overall UPy‐to‐polymer molar ratio was maintained at 1:1. The resulting products were designated PU‐IPDI‐UPy and PU‐HDI‐UPy, respectively. For comparison of the effects of hydrogen bonding interactions on the material performance, UPy chain extenders were fully or partially replaced by BDO, yielding PU‐IPDI‐BDO and PU‐IPDI‐UB, respectively (Supporting Information).

The successful incorporation of UPy into the PCL‐PDMS SPUE was verified by ^1^H NMR (Figures S1 and S2) and ATR‐FTIR (Figure S3). In the ^1^H NMR spectra of PU‐IPDI‐UPy, the characteristic signals of PDMS (H of Si‐CH_3_ at 0 ppm) and PCL (H of different CH_2_ at 1.4,1.6, 2.3 and 4.0 ppm, indicated in Figure S1) were observed, while additional peaks at 2.5–3.8 ppm and 4.2–5.0 ppm was assigned to methylene protons adjacent to amino ester and urea groups. Additionally, three signals at *δ* of 12.89, 11.90, and 10.06 ppm, corresponding to ─NH active hydrogen of UPy [33], were observed. The signals of UPy were also observed in ^1^H NMR spectra of PU‐HDI‐UPy (Figure S2). Gel permeation chromatography (GPC) analysis revealed that the SPUEs possess exceptionally high molecular weights, with number‐average molecular weights (*M*_n_) of 239 kg/mol for PU‐IPDI‐UPy and 149 kg/mol for PU‐HDI‐UPy, both markedly higher than that of PU‐IPDI‐BDO (∼94 kg/mol). This finding indicates that the incorporation of UPy segments enhances supramolecular interactions among the macromolecular chains, with PU‐IPDI‐UPy exhibiting the most pronounced degree of aggregation. The enhanced hydrogen‐bonding in PU‐IPDI‐UPy is attributed to the asymmetric substitution and cyclic structure of IPDI, which ensures optimal volume matching with UPy (illustrated in right side of Figure 1a). In the FTIR spectra, the C═O stretching vibration (*ν*_C═O_) can be deconvoluted into peaks at 1730, 1700, 1660, and 1640 cm^−1^ (Figure 1d,e), corresponding to free C═O, hydrogen bonded carbamate C═O, disordered urea C═O with a single hydrogen bond, and ordered urea C═O with bidentate hydrogen bonding, respectively [34]. By contrast, PU‐IPDI‐BDO presented only two peaks at 1730 and 1700 cm^−1^, attributed to free and hydrogen‐bonded carbamate C═O. PU‐IPDI‐UPy displayed substantially stronger hydrogen‐bonded C═O signals, with the fraction of hydrogen‐bonded C═O reaching 50.2% (Figure 1d), significantly exceeding those of PU‐HDI‐UPy (40.5%, Figure 1e) and PU‐HDI‐BDO (30.2%, Figure S4).

In the DSC curves from −70°C to 80°C, PU‐IPDI‐UPy only presented a glass transition temperature (*T*_g_) of PCL at −57°C [35], with no melting or crystallization observed during heating and cooling (Figure 1f). Therefore, the asymmetrical substitution of the cyclic chain extender IPDI, coupled with enhanced hydrogen‐bonding interaction, suppressed PCL crystallization. By contrast, PU‐HDI‐UPy displayed a melting peak at 43.5°C with an enthalpy of 24.5 J g^−1^ during the first heating process and displayed both cold crystallization and melting peaks during the second heating (Figure 1g). Similarly, PU‐IPDI‐BDO exhibited a melting peak at 44.6°C with an enthalpy of 35.9 J g^−1^ during the first heating cycle (Figure S5). The crystallization observed in the control groups (PU‐HDI‐UPy and PU‐IPDI‐BDO) stemmed from structural factors in that the linear HDI in PU‐HDI‐UPy reduced hydrogen bonding, and the substitution of UPy with BDO eliminated multiple hydrogen bonding, which in turn permitted PCL crystallization. Consequently, within the series, only PU‐IPDI‐UPy exhibited an amorphous state with strong hydrogen bonding interaction, which likely underpins its enhanced softness and potential strain‐stiffening characteristic, as will be elucidated in the following mechanical study. Furthermore, thermogravimetric analysis (TGA, Figure S6) showed that the decomposition temperatures of these PUs, defined at 5% weight loss, exceeded 250°C, reflecting their remarkable thermal stability and suitability for most practical applications.

### 2.2 Mechanical Performance and Strain‐Adaptive Stiffening of SPUE Thin Films

**FIGURE 2:**
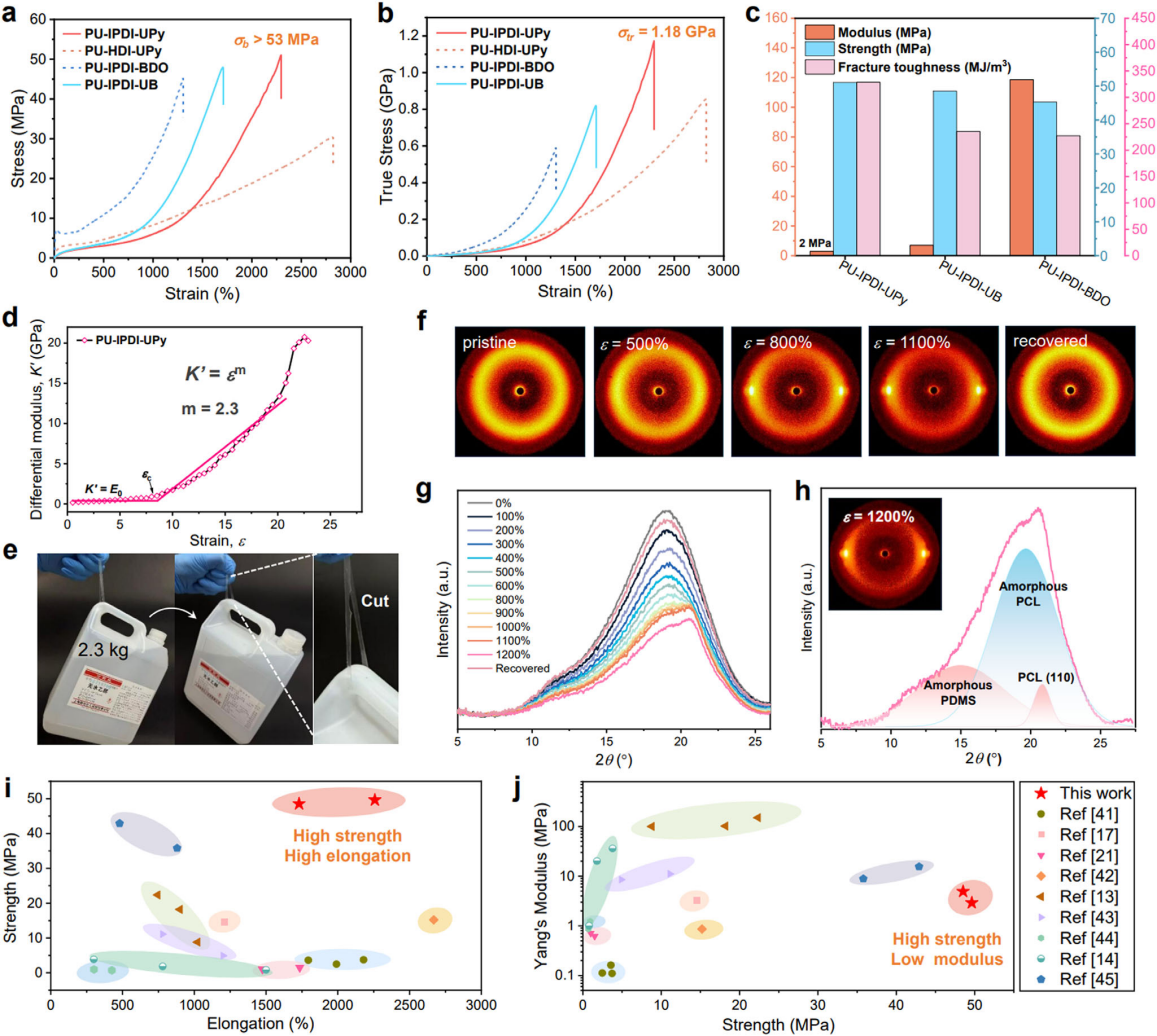
(a) Stress–strain curves of SPUE samples synthesized with different chain extenders. (b) Corresponding true stress–strain curves. (c) Comparison of the Yang's modulus, tensile strength, and stretchability of the indicated SPUE samples. (d) Differential modulus as a function of strain for PU‐IPDI‐UPy. (e) A PU‐IPDI‐UPy film suspends a 2.3 kg ethanol bottle and maintains the load even after cutting a notch at one side of the middle. (f) 2D WAXD patterns of PU‐IPDI‐UPy during in situ elongation at different strains. (g) 1D WAXD profiles of the PU‐IPDI‐UPy during the in situ stretching and after recovery. (h) Peak‐deconvoluted spectra of 1D WAXD pattern at 1200% elongation, with amorphous PDMS and PCL, as well as the (110) crystalline plane indicated. Various room‐temperature self‐healable elastomers are compared in terms of (i) strength versus elongation and (j) modulus versus strength, respectively [13, 14, 17, 21, 41, 42, 43, 44, 45].

Strain‐stiffening represents a fundamental biomechanical feature of natural skin, enabling skin to resist damage from excessive or unintended stretching while preserving its intrinsic softness. As shown in Figure 2a, the PU‐IPDI‐UPy exhibits a characteristic J‐shaped stress–strain profile, featuring initial soft elasticity followed by rapid strain‐stiffening. The material combines a skin‐like Young's modulus of ∼2.9 MPa (comparable to that of natural skin, 0.6–6 MPa) [2] with exceptional mechanical robustness, including a tensile strength of ∼51.1 MPa, elongation at break of 2297%, and fracture toughness of 311 MJ m^−3^. By contrast, PU‐IPDI‐BDO presents pronounced yielding behavior with a high Yong's modulus of 111 MPa, whereas PU‐HDI‐UPy shows an almost linear stress–strain response with an initial modulus of 12 MPa. The PU‐HDI‐UPy also exhibits an elongation at the beak of 2600%, underscoring the contribution of UPy units to energy dissipation and the enhancement of material toughness. Furthermore, the combination of UPy and BDO effectively tunes the modulus and the onset strain of stiffening. For example, PU‐IPDI‐UB displays a Yang's modulus of 6 MPa and a tensile strength of 48 MPa, with the strain‐stiffening initiating at a lower elongation than that observed for PU‐IPDI‐UPy. The true stress–strain curves (Figure 2b) reveal that PU‐IPDI‐UPy achieves the highest true strength of 1.17 GPa, approximately twice that of PU‐IPDI‐BDO (0.56 GPa). A comparative overview of the mechanical performance of PU‐IPDI‐UPy, PU‐IPDI‐UB, and PU‐IPDI‐BDO is provided in Figure 2c and Table S1. As corroborated by the FTIR and DSC results, the unique structural profile of PU‐IPDI‐UPy elastomer ensures significantly stronger hydrogen‐bonding interactions and a highly amorphous PCL phase compared to PU‐HDI‐UPy and PU‐IPDI‐BDO. This combination thereby gives rise to its characteristically low Young's modulus and marked strain‐stiffening behavior. Hence, the PU‐IPDI‐UPy was chosen for subsequent in‐depth study.

Based on the true stress–strain curve of PU‐IPDI‐UPy, the differential modulus *K*′ ≡ ∂σ/∂ε’ dependent on strain of PU‐IPDI‐UPy was calculated [36] and plotted in Figure 2d. At low strain, *K*′remains at a plateau modulus, but beyond a critical strain (*λ*_c_) ∼800%, *K*′ increases sharply, following *K*′ = ε^*m*^  where the exponent *m* is the stiffening index [37]. With a high elongation ability, PU‐IPDI‐UPy still exhibits a stiffening index of 2.3, indicating its pronounced strain‐stiffening capability. A 0.22 g PU‐IPDI‐UPy film can lift a 2.3 kg ethanol bottle approximately 10 000 times its own weight, and retains this load when cut with a blade (Figure 2e). Such exceptional performance attests to the material's superior strength and toughness arising from the synergistic interplay of strain‐stiffening and supramolecular reinforcement.

To elucidate the origin of the high strength and remarkable strain‐stiffening behavior of the elastomer, the morphological evolution of PU‐IPDI‐UPy under stretching was probed by in situ wide‐angle X‐ray diffraction (WAXD) and small‐angle X‐ray scattering (SAXS) experiments. Before 500% strain, the 2D WAXD patterns show an amorphous halo (Figure 2f). Upon further stretching beyond 800%, strain‐induced crystallization occurs, as evidenced by the sharp scattering spot along the equatorial direction. In the corresponding 1D WAXD profiles (Figure 2g,h), a new peak centered at 21.7° appeared in 1D WAXD profiles, attributed to the (110) plane of the crystallized PCL. According to the Scherrer equation, *D* = K*λ*/(*β*cos*θ*) [38], the crystalline particle size was calculated to be 3.5 nm, which is much smaller than that of typically melting‐crystallized PCL [39]. When the stress was released, the scattering pattern returned to be amorphous halo indicating the micro‐crystallization of PCL segments was reversible (Figure 2f). The SAXS profiles of PU‐IPDI‐UPy (Figure S7) before and after stretching show no discernible phase‐separation signal, indicating the absence of distinct microphase separation between PDMS and PCL. In contrast, PU‐HDI‐UPy exhibits pronounced crystallization signals in the SAXS profile even before stretching, and both phase separation and crystalline orientation become more evident upon deformation. The amorphous PU‐IPDI‐UPy with typical strain‐stiffening contributes to the outstanding mechanical performance.

According to the classic rubber elasticity theory, the relationship between the stress *σ* and elongation *λ* is described as follows [40],

$$\sigma =\frac{\rho RT}{M_{c}}\left(\lambda -\frac{1}{\lambda^{2}}\right)$$

where *λ* is the elongation ratio, *ρ* and *M*_c_ are the mass density and molecular weight of the segment between the crosslinking points of the SPUE. According to the Yang's modulus (E) at low elongation ε<30% and the statistical theory of rubber elasticity ($\frac{\rho RT}{M_{c}}=\frac{E}{3}\;$) [40], the molecular weight between crosslinks was determined to be 2700 g mol^−1^. This value is in close agreement with the molecular weight of PCL prepolymer, indicating that the IPDI‐UPy hard segments are nearly quantitatively aggregated into nanodomains that act as physical crosslinks. At strains below 800%, the stress exhibits a slow increase, consistent with the softness of amorphous PDMS and PCL segments, together with the effect of dynamic hydrogen‐bonding cross‐links, which collectively contribute to the soft elasticity. Beyond 800% strain, however, pronounced strain‐stiffening occurs, corresponding to an apparent decrease in *M*_c_ and the generation of additional cross‐linking points. In this regime, strain‐induced PCL microcrystals serve as additional physical crosslinks alongside entanglements, thereby reducing the effective *M*_c_ and giving rise to a sharp stress increase at high elongation.

Therefore, the synergistic combination of UPy and IPDI units in the hard segment imparts strong hydrogen‐bonding interactions while suppressing PCL crystallization, endowing the PU‐IPDI‐UPy elastomer with exceptional strength and toughness yet a low modulus. This molecular design effectively reconciles the typically antagonistic attributes of high strength and low stiffness. To highlight this advancement, a systematic comparison of strength, elongation, and modulus was performed between our elastomer and previously reported room‐temperature self‐healable PU elastomers [13, 14, 17, 21, 41, 42, 43, 44, 45]. As depicted in Figure 2i,j, the Ashby‐type plots of strength versus elongation and strength versus modulus demonstrate that our SPUE occupies a previously unattained regime, characterized by the concurrent realization of high strength, large elongation, and low modulus, outperforming most reported SPUEs. These results establish a new benchmark for soft‐yet‐robust supramolecular polyurethane elastomers.

### 2.3 Resilience and Dynamic Thermomechanical Response of PU‐IPDI‐UPy SPUE Films

**FIGURE 3:**
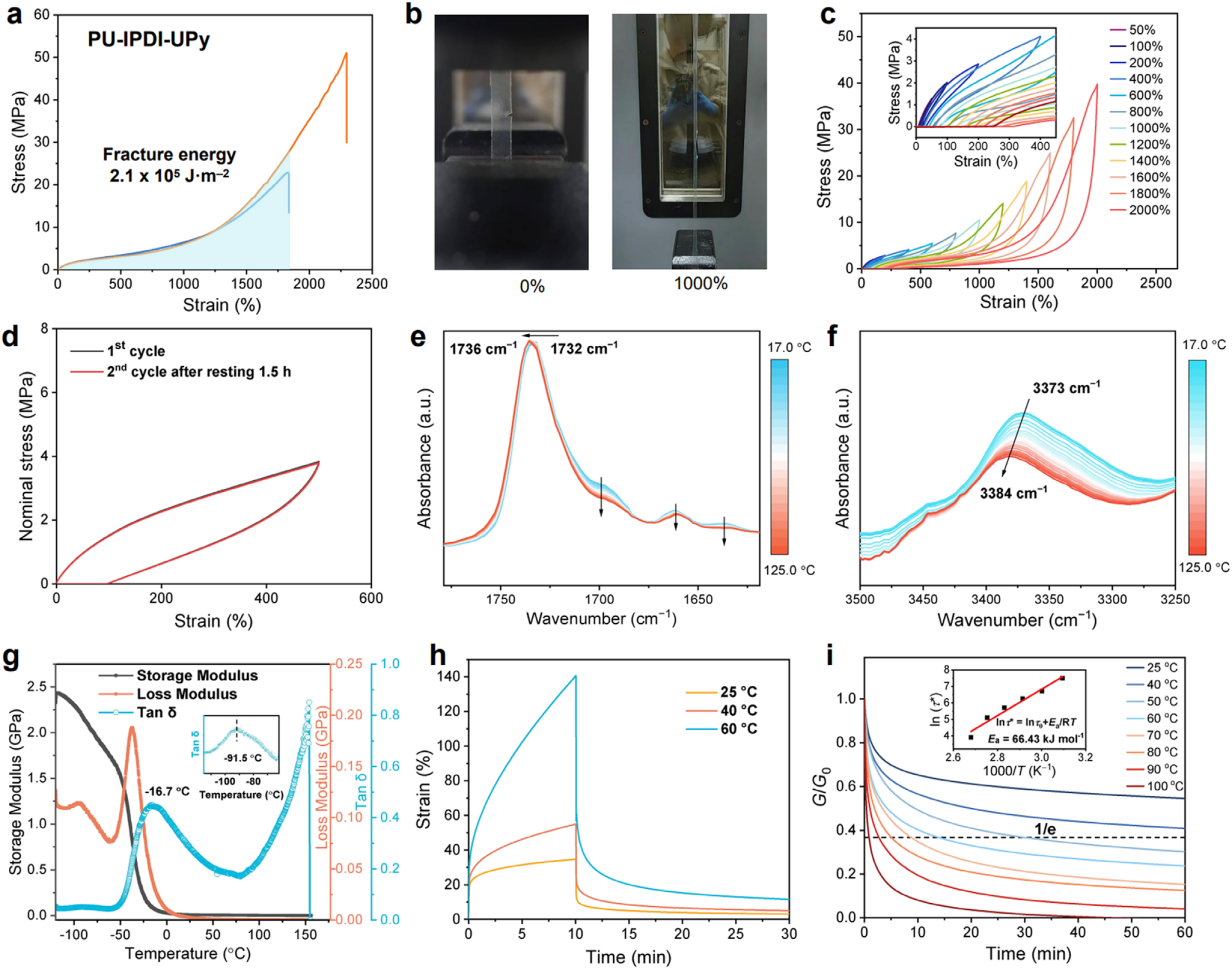
(a) Tensile stress–strain curves of the notched sample compared with the original one. (b) Photographs of the notched sample during the tensile experiment. (c) Cyclic loading‐unloading stress–strain curves of PU specimens under varying strain levels, 2D SAXS, and (d) that at 500% strain after resting for 2 h. (e, f) FTIR spectra recorded at variable temperatures from 17°C to 125°C. (g) DMA curves of PU‐IPDI‐UPy dependent on temperature. (h) Creep and creep recovery curves at different temperatures. (i) Stress relaxation curves at different temperatures, with the corresponding fitted curve of logarithmic relaxation time versus temperature.

Main text paragraph. We further assessed the tear resistance (notch sensitivity) and elastic resilience of the polyurethanes. As shown in Figure 3a, the tensile stress–strain curves of notched PU‐IPDI‐UPy SPUE films are presented along with the corresponding fracture energy determined using the Green–Smith method. PU‐IPDI‐UPy presented a high fracture energy of 2.1 × 10^5^ J m^−2^, relatively higher than other elastomers (Figure S8). During elongation, the single‐edge precrack progressively blunted (Figure 3b), and continued stretching resulted in increasing stress concentration at the crack tip.

Variable‐step, strain‐incremental cyclic tensile tests reveal that PU‐IPDI‐UPy, possessing the highest mechanical strength, also exhibits superior resilience. Its cyclic stress–strain curves display the characteristic ‘pine‐leaf’ hysteresis loops typical of rubber materials (Figure 3c). At 50% strain, the residual strain of PU‐IPDI‐UPy is approximately ∼11%. After a brief recovery period (4.3 s), a sustained positive stress emerges, indicating strain recovery, corresponding to a recovery rate of 8.7% s^−1^. This demonstrates the material's excellent elastic recovery capability. At strains below 800%, the residual strain remains below 50%, and even after multiple cycles under ultra‐high elongation (2000%), the residual strain stays below 300%. In contrast, PU‐HDI‐UPy exhibits deformation‐sensitive resilience (Figure S9), showing a residual strain 9.0% at 50% strain, and a rapid recovery rate of 12.4% s^−1^ comparable to that of PU‐IPDI‐UPy; however, as strain increases, the resilience of PU‐HDI‐UPy deteriorates markedly with residual strain reaching 1301% at 2000% strain. Movie S1 shows that the PU‐IPDI‐UPy film stretched to 280% can rapidly return to its original length. These results collectively highlight that PU‐IPDI‐UPy combines excellent instantaneous resilience at low strain, superior recovery at high strain, and efficient energy dissipation. Notably, in cyclic tensile tests at 500% strain, the PU‐IPDI‐UPy sample subjected to a 90 min rest before the second stretching shows nearly complete strain recovery (Figure 3d), as evidenced by the near‐perfect overlap of the cyclic curves.

Temperature‐dependent FTIR spectroscopy was employed to investigate the extensive dynamic H‐bond interactions in PU‐IPDI‐UPy during continuous heating (Figure 3e,f). Upon heating from 17°C to 125°C, the absorption bands corresponding to N─H and C═O groups exhibited clear shifts. The N─H stretching vibration underwent a blue shift from 3373 to 3384 cm^−1^, while the free C═O band showed a blue shift from 1732 to 1736 cm^−1^. Simultaneously, the intensity of hydrogen‐bonded C═O decreased markedly with increasing temperature. DMA modulus‐temperature scan results (Figure 3g) reveal that PU‐IPDI‐UPy exhibits two distinct glass transition temperatures (*T*_g_) in the loss modulus‐temperature curve, corresponding to the PDMS segment (−91.5°C) and the PCL segment (−16.7°C), respectively. The glass transition temperature of PDMS and PCL was also obvious in the tan*δ* curves. These dynamic *T*_g_ values are consistently higher than the corresponding values obtained in static DSC measurements. The storage modulus‐temperature curve also shows a plateau below −100°C, followed by two sharp drops associated with the onset of segment mobility in PDMS and PCL. As the temperature approached the upper limit of the DMA measurement, *E*’ remains consistently higher than *E*″, indicating predominantly elastic behavior. Above 90°C, a sharp increase in tan*δ* is observed, which can be ascribed to the H‐bond dissociation and the increased chain movement, in agreement with the FTIR results.

Creep and stress relaxation are essential viscoelastic responses that reveal the chain mobility and network dynamics of polymers, thereby underpinning their durability and energy‐dissipation capabilities. As shown in Figure 3h, PU‐IPDI‐UPy displays remarkable creep recovery across different temperatures under a constant stress of 1 MPa. At 25°C, the creep strain (*R*_c_) reached 19% within 10 min, with a recovery rate (*R*_rc_) of 91% within 2 min after the stress release. Increasing the temperature to 40°C and 60°C led to higher *R*_c_ values of 35% and 114%, respectively, while *R*_rc_ consistently remained above 91%. The temperature‐dependent enhancement in creep strain arises from the dynamic crosslinked network, which promotes chain mobility at elevated temperatures. Notably, the PU‐IPDI‐UPy maintains robust creep recovery due to its hierarchical hydrogen bonding architecture, wherein strongly dimerized UPy units form quadruple hydrogen bonds that dissipate energy and facilitate rapid network reformation. Accordingly, the stress relaxation behavior (Figure 3i) reveals progressively faster relaxation kinetics with increasing temperature. Arrhenius analysis of the relaxation process (inset of Figure 3i) yields an activation energy (*E*_a_) of 66.43 kJ mol^−1^, a typical value for supramolecular polyurethane elastomers, suggesting a moderate energy barrier for network reorganization. This thermally activated dynamic behavior endows PU‐IPDI‐UPy with an optimal combination of mechanical robustness and intrinsic self‐healing capability, ensuring reliable performance under service conditions.

### 2.4 Self‐Healing and Hydrophobic Properties of PU‐IPDI‐UPy and Its Conductive Composites

**FIGURE 4:**
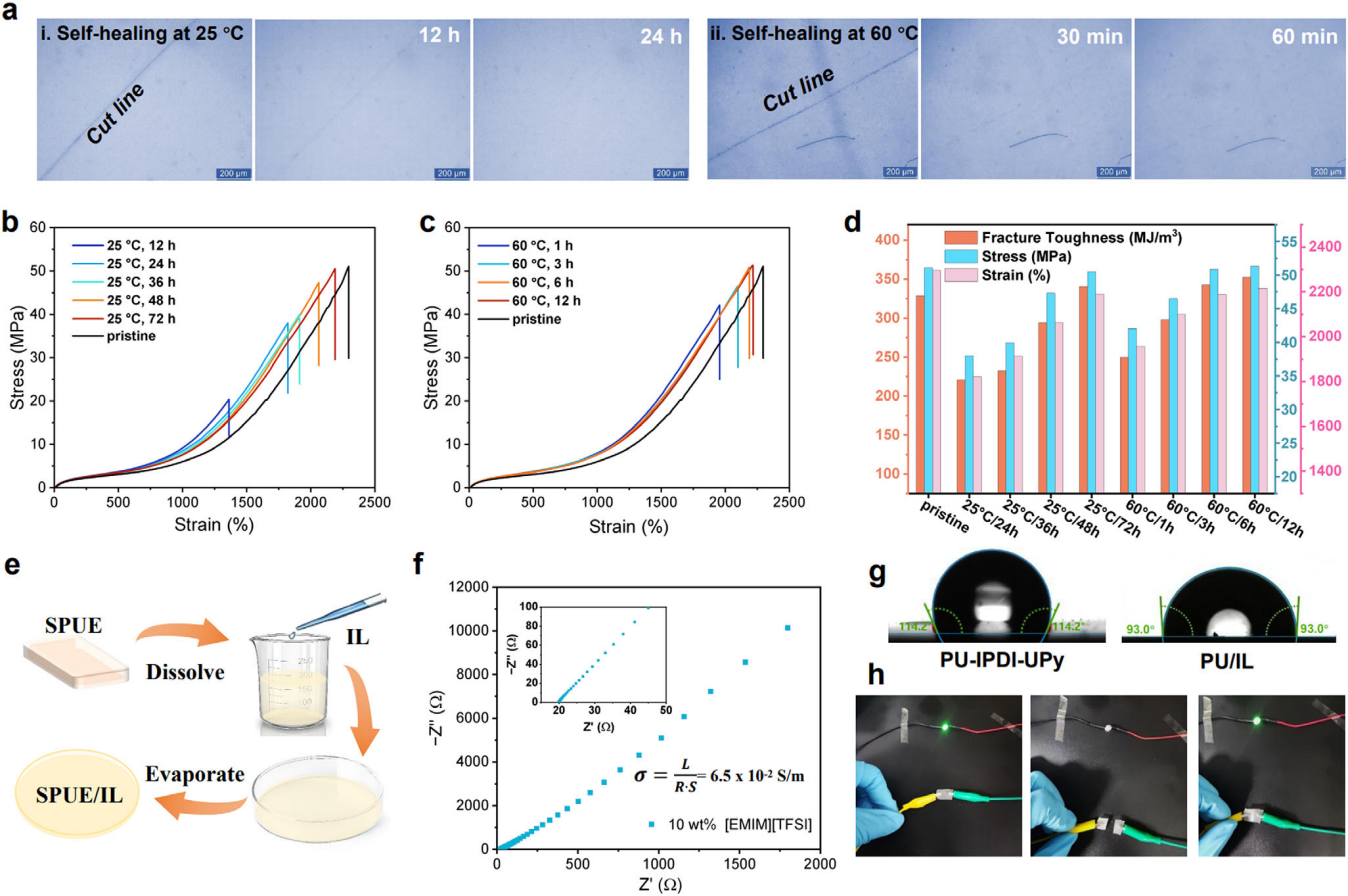
(a) Optical images showing the self‐healing process of scratched PU‐IPDI‐UPy films at room temperature and 60°C. (b, c) Stress–strain curves of the PU‐IPDI‐UPy films after self‐healing at room temperature and 60°C, respectively. (d) Comparison of tensile strength, fracture strain, and toughness of PU‐IPDI‐UPy healed at different conditions with the pristine sample. (e) Dissolving and remolding process of SPUE/IL composite. (f) Nyquist plot of SPUE/IL composite with the corresponding calculated ionic conductivity. (g) Water contact‐angle photographs of PU‐IPDI‐UPy films and the SPUE/IL composite. (h) Photographs showing that the conductive composite lights a bulb in an intact circuit, loses conductivity after being cut, and regains circuit function upon self‑healing.

Enhancing the repairability of polymeric materials is essential for extending service lifetime and promoting material sustainability. The self‐healing behavior of PU‐IPDI‐UPy was systematically evaluated through surface scratch and mechanical recovery tests. When a cross‐shaped scratch was introduced on both sides of the film, nearly complete healing occurred within 24 h at room temperature, while full recovery was achieved within 30 min at 60°C (Figure 4a). Mechanical recovery tests further validated the intrinsic self‐healing capability. At room temperature, the elastomer recovered approximately 90% of its original tensile properties within 48 h and achieved full restoration after 72 h (Figure 4b). In contrast, thermal treatment at 60°C accelerated the healing process, enabling ∼90% recovery within 3 h and complete restoration within 6 h (Figure 4c). These results demonstrate that PU‐IPDI‐UPy can fully restore its structural integrity under ambient or moderately elevated temperatures. Such rapid and efficient recovery can be attributed to the synergistic interplay of hierarchical hydrogen‐bonding interactions and flexible chain architecture, which collectively enable dynamic network reconstruction and rapid healing kinetics.

Moreover, the skin‐like softness and toughness of the PU‐IPDI‐UPy SPUEs, derived from the incorporation of biocompatible PDMS and PCL segments, render them promising candidates for artificial skin applications. To demonstrate this potential, an ionic skin prototype was fabricated using SPUE/[EMIM][TFSI] composite films prepared via solution blending followed by solvent evaporation (Figure 4e). When blending 10 wt.% ionic liquid, the films exhibited an ionic conductivity of 6.5 × 10^−2^ S m^−1^ (Figure 4f). Owing to the intrinsic hydrophobic nature of the PDMS segments and ionic liquid, the SPUE films were hydrophobic with a water contact angle of 114 ± 3° and the SPUE/IL composite films still displayed excellent hydrophobicity with water contact angles of 93 ± 2° (Figure 4g). The robust UPy‐mediated multiple hydrogen‐bonding PDMS‐PCL network ensured high resistance to moisture and moderate thermal fluctuations. The self‐healing capability of the composite film was then verified through electrical circuit integration (Figure 4h): upon severing, the circuit was interrupted, and the diode switched off; after reconnection, both structural integrity and electrical conductivity were restored, recovering the original luminance. Collectively, these demonstrations highlight the modularly engineered supramolecular elastomer as a multifunctional material that integrates strain‐stiffening mechanics, reliable conductivity, and intrinsic self‐healing, thereby offering substantial promise for next‐generation electronic skin combining tactile sensitivity with long‐term durability.

## 3 Conclusion

In summary, inspired by the mechanical adaptability of natural skin, a supramolecular polyurethane was designed by integrating soft PDMS, crystallizable PCL, and strongly associating UPy motifs via a chain‐extension strategy. The integration of volume‐matched IPDI and UPy chain extenders enhances hydrogen‐bonding crosslinking and ensures the reversible macrocyclization of PCL during elongation. The resulting material uniquely couples an ultralow modulus (3 MPa) with high strength (51 MPa) and room‐temperature self‐healing, effectively overcoming the intrinsic trade‐off between softness and robustness. The morphology evolution during stretching detected by the XRD indicated a 3.5 nm microcrystal of PCL induced during elongation. Cyclic tensile experiment revealed the energy dissipation and the outstanding resilience of the elastomer. The polymer segments exhibit excellent compatibility with the ionic liquid, preliminarily demonstrating their outstanding potential for high‐performance ionic skin applications. This work demonstrates that rational modulation of chain topology enhances strength and crack resistance while preserving strain‐stiffening and healability, providing a versatile strategy for designing long‐lived, skin‐like elastomers for next‐generation wearable electronics and artificial skin.

## 4 Experimental Section

### 4.1 Materials

PCL‐diols with a number‐average molecular weight (*M*_n_) of 2000 g mol^−1^ were synthesized by ring‐opening polymerization of ε‐caprolactone according to our previously reported procedure [35]. Ureidopyimidinone (UPy) was synthesized (Supporting Information) and used after drying. Hydroxyl terminated‐PDMS (*M*_n_ = 500 g/mol, purchased from Sigma–Aldrich), tetrahydrofuran (THF, AR, 99.5%, Greagent), dibutyltin dilaurate (DBTDL, RG, 95%+, Adamas), isophorone diisocyanate (IPDI, 99%, Aladdin), hexamethylene diisocyanate (HDI, 99%, Aladdin), 1,4‐butanediol (BDO, 99%, Aladdin), N, N‐dimethylformamide (AR, 99.5%, Chron Chemicals), dimethyl sulfoxide (DMSO, anhydrous, <50 ppm H_2_O, Adamas) were purchased and used.

### 4.2 Synthesis of SPUE

The target SPUEs were synthesized via a two‐step polymerization approach, as illustrated in Scheme S1. In a typical procedure for PU‐IPDI‐Upy, the dried PDMS and PCL diols at a fixed molar ratio of 1:4 were charged into a flame‐dried reactor and dried under vacuum at 80°C for 6 h. Under a nitrogen atmosphere at 60°C, a solution of isophorone diisocyanate (IPDI) (2.08 equiv. relative to total polymer) and a catalytic amount of dibutyltin dilaurate (DBTDL, 0.1 mol% of diols) in anhydrous THF was added. The reaction proceeded at 60°C for 12 h. Subsequently, an equimolar amount (relative to diols) of the chain extender 2‐ureido‐4[1H]‐pyrimidinone (UPy) in DMSO was introduced, and the chain extension continued at 60°C for another 24 h. The product was precipitated into ice n‐hexane to precipitate, filtered, washed thoroughly, and dried under vacuum at 40°C for 48 h to obtain the final elastomer. Control polymers were prepared following the same two‐step procedure as described for SPUE‐IPDI, with selective substitution of either the diisocyanate or the chain extender. Specifically, PU‐IPDI‐BDO was synthesized using IPDI and 1,4‐butanediol (BDO, dissolved in THF) as the chain extender, while PU‐HDI‐UPy was obtained by employing HDI with the UPy chain extender. All other reaction and workup conditions remained identical.

Polymer films were prepared by dissolving a predetermined amount of the synthesized PU product in tetrahydrofuran. The homogeneous solution was cast into a polytetrafluoroethylene (PTFE) mold and left to volatilize at room temperature. After complete solvent evaporation, the resulting film was further dried under vacuum to remove any residual solvent. Standard dumbbell‐shaped specimens for mechanical testing were then cut from the film using a precision die cutter.

To prepare SPUE/IL composite film, the PU‐IPDI‐UPy elastomer film was dissolved in THF (≈0.05 g/mL), and [EMIM][TFSI] ionic liquid was separately dissolved in THF (≈0.5 g/mL). The solutions were mixed at a PU‐to‐IL mass ratio of 10:1 under stirring, cast into a PTFE mold, and allowed to dry slowly at room temperature for 48 h. The resulting film was then vacuum‐dried at 60°C for 12 h.

### 4.3 Chemical Structure and Thermal Behavior Characterization

^1^H NMR spectra were recorded on a Bruker AVANCE III 400 MHz spectrometer at room temperature using CDCl_3_ as the solvent. Chemical shifts are reported in ppm relative to Si‐CH_3_ of PDMS (0 ppm). Attenuated total reflection Fourier transform infrared (ATR‐FTIR) spectroscopy was performed on a Thermo Scientific Nicolet iS50 spectrometer. The molecular weights and polydispersity indices (*Đ*) of the polymers were determined by gel permeation chromatography (GPC) using a Waters 1515 system with THF serving as the mobile phase at a flow rate of 1.0 mL min^−1^, and calibration was based on narrow‐dispersity polystyrene standards.

Thermogravimetric analysis (TGA) was performed using a NETZSCH TG 209F1 analyzer under a constant nitrogen flow. Samples (∼5 mg) were heated from 35°C to 800°C at a rate of 10°C min^−1^. The onset decomposition temperature (*T*_d_, onset) was determined as the temperature at which 5% mass loss occurred. Differential scanning calorimetry (DSC) measurements were carried out on a TA Instruments Q250 calorimeter. Specimens (∼4 mg) were subjected to consecutive heating/cooling cycles between −80°C and 150°C at a rate of 10°C min^−1^ under a nitrogen purge of 50 mL min^−1^.

### 4.4 Mechanical Property

The mechanical properties were evaluated at room temperature using an INSTRON 5967 universal testing machine. Dumbbell‐shaped specimens were stretched at a constant rate of 50 mm·min^−1^. Tensile strength, elongation at break, and Young's modulus were directly determined from the obtained stress–strain curves. The fracture toughness (*W*), representing the total energy dissipated prior to fracture, was calculated by integrating the area under the corresponding stress–strain curve.

(1) $$W=\int_{\varepsilon =0}^{\varepsilon =\varepsilon_{b}}\sigma d\varepsilon$$

where *σ* and *ε* are the engineering stress and strain, respectively, and *ε*_b_ is the strain at fracture. The fracture energy (*G*_c_) was then calculated using the relationship as follows:[46]

(2) $$G_{c}=\frac{6WC}{\sqrt{\lambda_{c}}}$$

where λ_C_ is the fracture stretch ratio of the notched specimen, *C* is the length of the notch, and *W* is the energy calculated by integrating the stress–strain curve of the unnotched sample strain from 0 to *λ*_C_.

### 4.5 Nanostructure Characterization

To probe the molecular‐level structural evolution during deformation, the nanostructure and orientation development within the SPUE films were characterized in real time by in situ wide‐angle x‐ray diffraction (WAXD) measurements using a Bruker D8 Discover diffractometer equipped with an IµS microfocus Cu Kα source (λ = 0.1542 nm) operating at 50 kV and 1000 µA. A VÅNTEC‐500 2D detector (pixel size: 136 × 136 µm^2^) was employed. Measurements were conducted in transmission mode with a sample‐to‐detector distance of 100 mm and a beam spot size of 0.5 mm. For in situ structural analysis during deformation, the polymer films were mounted on a Linkam TST350 tensile stage integrated with the diffractometer. Dumbbell‐shaped specimens (gauge dimensions: 6 mm length × 4 mm width) were stretched at a constant rate of 3 mm min^−^^1^. 2D WAXD patterns were acquired at 5‐min intervals throughout the entire tensile process.

2D Small Angle X‐ray Scattering (2D‐SAXS) tests of PU were carried out on a Bruker Nanostar SAXS instrument using Cu Kα radiation at a wavelength of 0.154 nm. The operating voltage and current were 50 kV and 0.60 mA, respectively.

### 4.6 Dynamic Thermomechanical Analysis (DMA) Measurement

DMA measurements were conducted on a TA Q800 analyzer in tensile mode. Rectangular film samples were tested from −150°C to 150°C at a heating rate of 5°C min^−^^1^, with a frequency of 1 Hz and a strain amplitude of 0.1% under a nitrogen atmosphere. The temperature‐dependent storage modulus (E′), loss modulus (E″), and loss tangent (tan δ) were recorded.

Stress relaxation was measured on a TA Q800 DMA across 25°C–100°C. At each temperature, a constant strain of 50% was applied, and stress decay was recorded. The normalized relaxation modulus (*G*(t)/*G*_0_) was plotted versus time, and the activation energy (*E*_a_) was calculated using the Arrhenius equation:

(3) $$ln\tau^{*}=ln\tau_{0}+\frac{E_{a}}{RT}$$

where *τ* ^*^ is the characteristic relaxation time at temperature* T*, τ_0_ is the pre‐exponential factor, R is the universal gas constant (8.314 J·mol^−1^·K^−1^), and *T* is the test temperature in Kelvin.

Creep behavior was characterized using the creep compliance mode of the same DMA Q800 instrument. Tests were conducted at 25°C, 40°C, and 60°C, respectively. At each temperature, a constant tensile stress in the range of 0.5–1.0 MPa—determined from prior tensile stress–strain curves—was applied and maintained for 10 min, followed by a recovery period of 20 min under zero load. The instantaneous strain upon loading was denoted as *ε*_1_, the strain at the end of the creep period as *ε*_2_, and the residual strain after recovery as* ε*_3_. The creep rate (*R*_c_) and creep recovery rate (*R*_rc_) were calculated as follows:

(4) $$R_{c}=\frac{\varepsilon_{2}-\varepsilon_{1}}{\varepsilon_{1}}\times 100\%$$

(5) $$R_{rc}=\frac{\varepsilon_{2}-\varepsilon_{3}}{\varepsilon_{2}}\times 100\%$$

### 4.7 Self‐Healing Performance Evaluation

The healing capability of the SPUE materials was assessed through both visual inspection and mechanical recovery. The autonomous healing behavior was visually demonstrated using a scratch‐recovery test. A controlled scratch was introduced on the film surface with a sharp blade, and the sample was then placed at room temperature or on a 60°C hot plate. The progressive closure of the scratch was monitored in real time using a Leica DM4P polarizing optical microscope. For mechanical evaluation, tensile specimens were bisected and subsequently rejoined at room temperature or at 60°C for a certain period under slight manual pressure. The mechanical property recovery was quantified by re‐testing the healed specimens following the same tensile protocol detailed in Section 4.4.

### 4.8 Electrochemical Impedance Spectroscopy (EIS) and Hydrophobicity Measurements

Electrochemical impedance spectroscopy (EIS) was measured on a CHI 760E electrochemical workstation in the frequency range of 1–10^5^ Hz. The electrical conductivity (*σ*) of the sample was calculated using σ=L/(R·A), where L is the length between electrodes, *R* is the real part of the impedance (*Z*′) obtained from the Nyquist plot, and A is the cross‐sectional area of the specimen.

Static water contact angles were measured at ambient temperature using an optical contact angle goniometer (DSA25, KRÜSS GmbH, Germany). Deionized water droplets (2 µL) were gently deposited onto the sample surface using an automated syringe. The contact angle of each droplet was determined from the side‑view image via the Young‑Laplace fitting method. For each sample, at least five independent measurements were performed on different surface locations, and the average value with standard deviation is reported.

## Conflicts of Interest

The authors declare no conflicts of interest.

## Supporting information




**Supporting File**: advs74910‐sup‐0001‐SuppMat.docx.

## Data Availability

The data that support the findings of this study are available from the corresponding author upon reasonable request.

## References

[1] M. Wang , Y. Luo , T. Wang , et al., “Artificial Skin Perception,” Advanced Materials 33 (2021): 2003014, 10.1002/adma.202003014. 32930454

[2] A. M. Kalra , A. Lowe , and A. M. Al‐Jumaily , “Mechanical behaviour of skin: A Review,” Journal of Material Science & Engineering 5 (2016): 1000254.

[3] G. Holzapfel , “Biomechanics of Soft Tissue,” The Handbook of Materials Behavior Models, (Academic Press, 2001), 1049–1063.

[4] C. Xu , S. A. Solomon , and W. Gao , “Artificial Intelligence‐Powered Electronic Skin,” Nature Machine Intelligence 5 (2023): 1344–1355, 10.1038/s42256-023-00760-z. PMC10868719 38370145

[5] J. Guo , C. Xiang , A. Conn , and J. Rossiter , “All‐Soft Skin‐Like Structures for Robotic Locomotion and Transportation,” Soft Robotics 7 (2019): 309–320, 10.1089/soro.2019.0059. PMC7301317 31730389

[6] X. Lyu , H. Zhang , S. Shen , Y. Gong , P. Zhou , and Z. Zou , “Multi‐Modal Sensing Ionogels With Tunable Mechanical Properties and Environmental Stability for Aquatic and Atmospheric Environments,” Advanced Materials 36 (2024): 2410572, 10.1002/adma.202410572. 39292213

[7] N. Zhou , T. Cui , Z. Lei , and P. Wu , “Bioinspired Learning And Memory In Ionogels Through Fast Response And Slow Relaxation Dynamics Of Ions,” Nature Communications 16 (2025): 4573, 10.1038/s41467-025-59944-3. PMC12084631 40379652

[8] J. Ince , K. Prasad , K. Subhani , A. Duffy , and N. Salim , “Liquid Crystalline Elastomers As Artificial Muscles And Flexible Actuators For Robotics/Hybrid Engineered Machinery,” Advanced Composites and Hybrid Materials 7 (2024): 186, 10.1007/s42114-024-00988-2.

[9] M. Vatankhah‐Varnosfaderani , A. N. Keith , Y. Cong , et al., “Chameleon‐Like Elastomers With Molecularly Encoded Strain‐Adaptive Stiffening And Coloration,” Science 359 (2018): 1509–1513, 10.1126/science.aar5308. 29599240

[10] S. Nakagawa , D. Aoki , Y. Asano , and N. Yoshie , “Module‐Assembled Elastomer Showing Large Strain Stiffening Capability and High Stretchability,” Advanced Materials 35 (2023): 2301124, 10.1002/adma.202301124. 36929528

[11] S. Lin , J. Liu , X. Liu , and X. Zhao , “Muscle‐Like Fatigue‐Resistant Hydrogels By Mechanical Training,” Proceedings of the National Academy of Sciences 116 (2019): 10244–10249, 10.1073/pnas.1903019116. PMC6535018 31068458

[12] J.‐H. So , A. S. Tayi , F. Güder , and G. M. Whitesides , “Stepped Moduli in Layered Composites,” Advanced Functional Materials 24 (2014): 7197–7204, 10.1002/adfm.201401548.

[13] H. Jiang , T. Yan , M. Cheng , et al., “Autonomous Self‐Healing And Superior Tough Polyurethane Elastomers Enabled By Strong And Highly Dynamic Hard Domains,” Materials Horizons 12 (2025): 599–607, 10.1039/D4MH01129E. 39503624

[14] Y. Chen , A. M. Kushner , G. A. Williams , and Z. Guan , “Multiphase Design Of Autonomic Self‐Healing Thermoplastic Elastomers,” Nature Chemistry 4 (2012): 467–472, 10.1038/nchem.1314. 22614381

[15] Y. Yanagisawa , Y. Nan , K. Okuro , and T. Aida , “Mechanically Robust, Readily Repairable Polymers Via Tailored Noncovalent Cross‐Linking,” Science 359 (2018): 72–76, 10.1126/science.aam7588. 29242235

[16] S.‐M. Lee , E. Pippel , U. Gösele , et al., “Greatly Increased Toughness of Infiltrated Spider Silk,” Science 324 (2009): 488–492, 10.1126/science.1168162. 19390040

[17] L. Zhang , Z. Liu , X. Wu , et al., “A Highly Efficient Self‐Healing Elastomer With Unprecedented Mechanical Properties,” Advanced Materials 31 (2019): 1901402, 10.1002/adma.201901402. 30977571

[18] J. Wu , F. Zeng , Z. Fan , S. Xuan , Z. Hua , and G. Liu , “Hierarchical Hydrogen Bonds Endow Supramolecular Polymers With High Strength, Toughness, and Self‐Healing Properties,” Advanced Functional Materials 34 (2024): 2410518, 10.1002/adfm.202410518.

[19] X. Yan , Z. Liu , Q. Zhang , et al., “Quadruple H‐Bonding Cross‐Linked Supramolecular Polymeric Materials as Substrates for Stretchable, Antitearing, and Self‐Healable Thin Film Electrodes,” Journal of the American Chemical Society 140 (2018): 5280–5289, 10.1021/jacs.8b01682. 29595956

[20] J. Lai , M. Xie , Q. Zhao , C. Zhang , Z. Wang , and H. Xia , “Underwater Non‐Contact and Ultra‐Fast Adaptive Self‐Healing Elastomers Based on Programmable Dissociation of Dynamic Bonds,” Advanced Functional Materials 35 (2025): 2415732, 10.1002/adfm.202415732.

[21] J. Kang , D. Son , G.‐J. N. Wang , et al., “Tough and Water‐Insensitive Self‐Healing Elastomer for Robust Electronic Skin,” Advanced Materials 30 (2018): 1706846, 10.1002/adma.201706846. 29424026

[22] Z. Feng , M. Xie , J. Lai , Z. Wang , and H. Xia , “Stereochemistry‐Tuned Hydrogen‐Bonding Synergistic Covalent Adaptable Networks: Towards Recycled Elastomers with Excellent Creep‐Resistant Performance,” Angewandte Chemie International Edition 64 (2025): 202423712. 10.1002/anie.202423712 39834149

[23] R. H. Aguirresarobe , S. Nevejans , B. Reck , et al., “Healable and Self‐Healing Polyurethanes Using Dynamic Chemistry,” Progress in Polymer Science 114 (2021): 101362, 10.1016/j.progpolymsci.2021.101362.

[24] J. Tian , Z. Zhou , X. Miao , et al., “Supertough, Resilient, and Healable Thermoplastic Poly (Urethane Urea) Elastomers by Dense Packing of Hydrogen‐Bonding Arrays,” Advanced Functional Materials 36 (2025): 04882.

[25] P. Y. W. Dankers , M. C. Harmsen , L. A. Brouwer , M. J. A. Van Luyn , and E. W. Meijer , “A Modular And Supramolecular Approach To Bioactive Scaffolds For Tissue Engineering,” Nature Materials 4 (2005): 568–574, 10.1038/nmat1418. 15965478

[26] M. Guo , L. M. Pitet , H. M. Wyss , M. Vos , P. Y. W. Dankers , and E. W. Meijer , “Tough Stimuli‐Responsive Supramolecular Hydrogels With Hydrogen‐Bonding Network Junctions,” Journal of the American Chemical Society 136 (2014): 6969–6977, 10.1021/ja500205v. 24803288

[27] J. Verjans and R. Hoogenboom , “Supramolecular Polymer Materials Based On Ureidopyrimidinone Quadruple Hydrogen Bonding Units,” Progress in Polymer Science 142 (2023): 101689, 10.1016/j.progpolymsci.2023.101689.

[28] K. Yang , Q. Li , S. Tian , et al., “Highly Stretchable, Self‐Healing, and Sensitive E‐Skins at −78°C for Polar Exploration,” Journal of the American Chemical Society 146 (2024): 10699–10707, 10.1021/jacs.4c00541. 38518116

[29] Y. Song , Y. Liu , T. Qi , and G. L. Li , “Towards Dynamic but Supertough Healable Polymers Through Biomimetic Hierarchical Hydrogen‐Bonding Interactions,” Angewandte Chemie International Edition 57 (2018): 13838–13842, 10.1002/anie.201807622. 30144244

[30] D. J. M. van Beek , A. J. H. Spiering , G. W. M. Peters , K. te Nijenhuis , and R. P. Sijbesma , “Unidirectional Dimerization and Stacking of Ureidopyrimidinone End Groups in Polycaprolactone Supramolecular Polymers,” Macromolecules 40 (2007): 8464–8475, 10.1021/ma0712394.

[31] D. K. Hohl , A.‐C. Ferahian , L. Montero de Espinosa , and C. Weder , “Toughening of Glassy Supramolecular Polymer Networks,” ACS Macro Letters 8 (2019): 1484–1490, 10.1021/acsmacrolett.9b00710. 35651179

[32] Z.‐X. Fei , C. Yin , J.‐R. Sun , L. Yuan , and L.‐Y. Shi , “Healable and Recyclable Multiblock Polyurethanes With Mechanical Performance Tailorability Based On Hierarchical Phase Separation And Dynamic Bond Interaction,” Polymer 289 (2023): 126467, 10.1016/j.polymer.2023.126467.

[33] X. Wang , C. Xu , N. Xu , et al., “Design, Synthesis, and Characterization of Semiconducting Polymers Incorporating a Ring‐Like Ureidopyrimidinone (UPy) Quadruple Hydrogen Bonding Structure,” Macromolecules 56 (2023): 5369–5380, 10.1021/acs.macromol.3c00443.

[34] S. Billa , P. Vislavath , J. Bahadur , et al., “Imparting Reprocessability, Quadruple Shape Memory, Self‐Healing, and Vibration Damping Characteristics to a Thermosetting Poly(urethane‐urea),” ACS Applied Polymer Materials 5 (2023): 3079–3095, 10.1021/acsapm.3c00225.

[35] Z.‐Y. Xu , L. Li , L.‐Y. Shi , K.‐K. Yang , and Y.‐Z. Wang , “Effect of Self‐Nucleation and Stress‐Induced Crystallization on the Tunable Two‐Way Shape‐Memory Effect of a Semicrystalline Network,” Macromolecules 55 (2022): 5104–5114, 10.1021/acs.macromol.2c00575.

[36] J. Luo , S. Li , J. Xu , et al., “Biomimetic Strain‐Stiffening Hydrogel With Crimped Structure,” Advanced Functional Materials 31 (2021): 2104139, 10.1002/adfm.202104139.

[37] M. Jaspers , M. Dennison , M. F. J. Mabesoone , F. C. MacKintosh , A. E. Rowan , and P. H. J. Kouwer , “Ultra‐Responsive Soft Matter From Strain‐Stiffening Hydrogels,” Nature Communications 5 (2014): 5808, 10.1038/ncomms6808. PMC4275588 25510333

[38] N. S. Murthy , Polymer Morphology: Principles, Characterization, and Processing (John Wiley & Sons, 2016): 14–36, 10.1002/9781118892756.

[39] H. Chen , Z. Sun , K. Lu , J. Liu , C. He , and D. Mao , “Negative Enthalpy Variation Drives Rapid Recovery in Thermoplastic Elastomer,” Advanced Materials 36 (2024): 2311332, 10.1002/adma.202311332. 38108494

[40] U. W. Gedde and M. S. Hedenqvist , Fundamental Polymer Science, edited by U. W. Gedde and M. S. Hedenqvist (Springer International Publishing, 2019), 75–111.

[41] Y. Tian , Y. Wei , M. Wang , et al., “Ultra‐Stretchable, Tough, And Self‐Healing Polyurethane With Tunable Microphase Separation For Flexible Wearable Electronics,” Nano Energy 139 (2025): 110908, 10.1016/j.nanoen.2025.110908.

[42] X. Wang , Y.‐L. Wang , X. Yang , Z. Lu , Y. Men , and J. Sun , “Skin‐Inspired Healable Conductive Elastomers With Exceptional Strain‐Adaptive Stiffening and Damage Tolerance,” Macromolecules 54 (2021): 10767–10775, 10.1021/acs.macromol.1c01976.

[43] M. A. Mohamed , C. L. Rumsey , M. Trebbin , and S. T. Andreadis , “Multifunctional Polyurethane Networks Combining Strength, Toughness, and Fast Autonomous Self‐Healing via the Synergy of Multiple Dynamic Bonds,” Chemistry of Materials 35 (2023): 6332–6345, 10.1021/acs.chemmater.3c00914. PMC13568685 42730383

[44] H. Ying , Y. Zhang , and J. Cheng , “Dynamic Urea Bond For The Design Of Reversible And Self‐Healing Polymers,” Nature Communications 5 (2014): 3218, 10.1038/ncomms4218. PMC4438999 24492620

[45] F. Sun , L. Liu , T. Liu , et al., “Vascular Smooth Muscle‐Inspired Architecture Enables Soft Yet Tough Self‐Healing Materials For Durable Capacitive Strain‐Sensor,” Nature Communications 14 (2023): 130, 10.1038/s41467-023-35810-y. PMC9829674 36624140

[46] H. W. Greensmith , “Rupture of Rubber. X. The Change In Stored Energy On Making A Small Cut In A Test Piece Held In Simple Extension,” Journal of Applied Polymer Science 7 (1963): 993–1002, 10.1002/app.1963.070070316.

