# Supplementary material for: Dynamic Supramolecular Polyurethane Elastomers Enabling Bioinspired Strain‐Adaptive Stiffening to Resolve the Softness–Strength Paradox
Source: Adv Sci (Weinh). 2026 Mar 20;13(31):e74910. doi: 10.1002/advs.74910 (PMC13588028; doi:10.1002/advs.74910)
Supplement: Supplementary file 1 [file ADVS-13-e74910-s001.docx]

**Supporting Information**

Dynamic Supramolecular Polyurethane Elastomers Enabling Bioinspired Strain-Adaptive Stiffening to Resolve the Softness–Strength Paradox

Haoming Zou,^[a]^ Zhi-Xiong Fei,^[a]^ Zhao Yang,^[a]^ Ke-Ke Yang,^[b]^ Dong-Xu Yang,*^[c]^ Ling-Ying Shi*^[a]^

[a] *College of Polymer Science and Engineering, State Key Laboratory of Polymer Materials Engineering, Sichuan University, Chengdu 610065, China.*
E-mail: shilingying@scu.edu.cn, ORCID ID (Ling-Ying Shi): 0000-0002-6620-7878

[b] *The Collaborative Innovation Center for Eco-Friendly and Fire-Safety Polymeric Materials (MoE), College of Chemistry, Sichuan University, Chengdu 610064, China*

[c] State Key Laboratory of Optical Field Manipulation Science and Technology, Institute of Optics and Electronics, Chinese Academy of Sciences, Chengdu, 610209, China.

E-mail: dxyang@ioe.ac.cn, ORCID ID (Dong-Xu Yang): 0000-0002-4753-1154

*** To whom correspondence should be addressed. Email: [shilingying@scu.edu.cn](mailto:shilingying@scu.edu.cn)

**Synthesis of 5-(2-Hydroxyethyl)-6-methyl-2-amino-4[1H] pyrimidinone (UPy)**

The synthesis of 5-(2-hydroxyethyl)-6-methyl-2-amino-4[1H] pyrimidinone (UPy) was performed according to a modified literature procedure. Specifically, the monomer α-acetyl-γ-butyrolactone (12.83 g, 0.1 mol), guanidine carbonate (9.01 g, 0.05 mol), and triethylamine (10.63 g, 0.105 mol) were sequentially added into a 250 mL three-necked round-bottom flask. Anhydrous ethanol (~100 mL) was introduced, and the mixture was stirred until complete dissolution. The reaction was conducted under reflux at 80 °C for 12 h using a spherical condenser. After approximately 10 h, no further increase in the white precipitate was observed. The reaction mixture was filtered directly, and the collected solid was washed alternately with ethanol and deionized water three times. The crude product was then suspended in water, and the pH was adjusted to approximately 6.5 using dilute hydrochloric acid under stirring. The solid was filtered again and subjected to another three cycles of alternate washing with ethanol and water. The final product was obtained as a pure white solid, which was subsequently dried under vacuum at 45 °C for 24 hours and stored in a desiccator for future use.

**Scheme S1**. Synthesis route of PU-IPDI-UPy, PU-HDI-UPy,PU-IPDI-BDO and PU-IPDI-UB. It is noted that when R_3_= (CH_2_)_6_, the elastomer was PU-HDI-UPy; when R_3_= IPDI, the elastomer was PU-IPDI-UPy,

**Figure S1**. ^1^H NMR spectrum of PU-IPDI-UPy, including peak attribution.

**Figure S2**. (a) ^1^H NMR spectra of PU-IPDI-UPy, PU-HDI-UPy and PU-IPDI-BDO with that of PDMS and PCL prepolymers, and (b) the enlarged one in the range of the hydrogen-bonded H of UPy.

**Figure S3**. FT-IR spectra of PU-IPDI-UPy, PU-HDI-UPy, PU-IPDI-BDO and PU-IPDI-UB with the enlarged one in the range of free and hydrogen-bonded C=O.

**Figure S4.** Deconvoluted FI-IR spectra of the C=O absorption of PU-IPDI-BDO.

**Figure S5.** (a-b) DSC curves of PU-IPDI-BDO and PU-IPDI-UB with the indication of glass transition and melting temperatures. (c) Comparison of *T*_g_ and crystallinity of SPUE samples with different chain extenders.

**Figure S6.** Thermogravimetric curves and the corresponding differential thermogravimetric (DTG) curves.

**Figure S7.** SAXS patterns of (a) of PU-IPDI-UPy, (b) PU-HDI-UPy and (c) PU-IPDI-BDO before and after stretching of 800%.

**Figure S8**. Tensile curves of the notched samples compared with the original one for (a) PU-HDI-UPy, (b) PU-IPDI-BDO and (c) PU-IPDI-UB.

**Figure S9**. Cyclic tensile stress–strain curves (a) PU-HDI-UPy, (b) PU-IPDI-BDO measured at various elongation ratios.

**Table S1.**  Mechanical strength, Yang’s modulus, elongation at break and fracture energy of different SPUEs.

| Sample | Tensile strength (MPa) | Young’s modulus (MPa) | Strain at break  (%) | Fracture toughness (MJ·m^-3^) |
| --- | --- | --- | --- | --- |
| PU-IPDI-UPy | 51.06 ± 2.5 | 2.99 ± 0.3 | 2297 ± 120 | 328.75 |
| PU-HDI-UPy | 30.39 ± 1.5 | 10.95 ± 1.0 | 2823 ± 90 | 383.62 |
| PU-IPDI-BDO | 45.30 ± 2.1 | 118.60 ± 3.0 | 1305 ± 48 | 227.22 |
| PU-IPDI-UB | 48.53 ± 2.3 | 7.06 ± 0.5 | 1730 ± 56 | 235.43 |
